# Association Between Allergic Rhinitis and Asthma in Adults With Loss of Interest in Sex

**DOI:** 10.7759/cureus.36823

**Published:** 2023-03-28

**Authors:** Norma A Pulido-Guillen, Jaime Morales-Romero, Martín Bedolla-Barajas, Tonantzin I Bedolla-Pulido, Claudia E Jiménez-Carrillo, Kevin J Arellano-Arteaga, Martin Robles-Figueroa

**Affiliations:** 1 Clinical Psychology, Center for Psychological Attention, Guadalajara, MEX; 2 Epidemiology and Public Health, Institute of Public Health, Veracruzana University, Xalapa, MEX; 3 Allergy and Clinical Immunology, Civil Hospital of Guadalajara "Dr. Juan I. Menchaca", Guadalajara, MEX; 4 Otolaryngology, University of Guadalajara, Guadalajara, MEX; 5 Allergy and Immunology, Regional Hospital ¨Dr. Valentín Gómez Farías" Institute of Social Security for Government Workers, Zapopan, MEX; 6 Internal Medicine, Civil Hospital of Guadalajara "Dr. Juan I. Menchaca", Guadalajara, MEX

**Keywords:** prevalence, sex, sexual dysfunction, asthma, allergic rhinitis

## Abstract

Background. The alterations of sexual desire in allergy respiratory diseases have seldom been analyzed. This paper aims to evaluate the association of allergic rhinitis and asthma among adults with the loss of interest in sex.

Methods. Through a cross-sectional study, we compared three groups of subjects: one with patients with allergic rhinitis, another with allergic asthma, and a control group. The loss of interest in sex was assessed with the Beck Depression Inventory-II, which includes a scale that evaluates this characteristic.

Results. The prevalence of loss of interest in sex in patients with allergic asthma, allergic rhinitis, and controls was 48.8%, 39.5%, and 20.2%, respectively. In multivariate models, a positive association between a loss of interest in sex and allergic asthma (OR =2.4, p =0.005) and allergic rhinitis (OR =2.1, p =0.03) was found independently. In both models, other associated factors included being female (p <0.001) and living as a couple (p <0.001). In contrast, no significant association was found with age (≥40 years), smoking, alcohol consumption, physical activity, or excess weight.

Conclusion. Loss of interest in sex is highly prevalent and is notoriously associated with allergic respiratory diseases; similarly, it is associated with living as a couple and being female.

## Introduction

Almost 340 and 400 million people worldwide suffer from allergic asthma [1] and rhinitis [2], respectively, two of the most common and chronic respiratory diseases. These diseases affect several aspects of the patient’s quality of life, including human sexuality. Regarding the five phases of sexual response (desire, excitation, plateau, orgasm, and resolution), previous studies have focused on the influence of allergic diseases in the late phases; however, studies about the impacts of the diseases in the initial phase are scarce. Shah and Sircar were the first to report the association between allergic asthma or rhinitis and sexual excitement [3]. Additionally, the impact of asthma on sexual life has been assessed recently in Spain, where 276 patients with asthma were compared to a control group; women with asthma were affected in all phases, while men were affected only in the phase of desire [4]. Allergic rhinitis symptoms impact sexual activity [5], causing men and women to have low scores on specialized questionnaires such as the International Index of Erectile Dysfunction or the Female Sexual Function Index, compared to men and women without allergic rhinitis [6]. Meaningful population studies point out two relevant results: first, losing interest in sex prevails in 13% to 43% of the adult population, and second, this prevalence is much higher in women than in men [7-9]. However, due to the limited knowledge related to these allergic diseases and the extent of this problem, and since the studies that have included patients with asthma do not differentiate between those with and without allergies, the main goal of the present study was to investigate whether the loss of interest in sex was associated with rhinitis or allergic asthma in adults.

## Materials and methods

Study design and patients

This was a cross-sectional study where three groups were compared: patients with allergic asthma (n = 164 s), patients with allergic rhinitis (n = 129), and a control group (n = 109).

Allergic asthma and allergic rhinitis patients were consecutively recruited from November 2018 to October 2019; subjects included were >18 years. Excluded subjects were those who had suffered from the death of a close relative during the previous six months, those using systemic steroids for a month, and those with diabetes mellitus, arterial hypertension, rheumatoid arthritis, rash, or renal insufficiency, and pregnant or nursing women.

Apart from an interest in sex, personal history of alcohol or cigarette consumption, as well as physical activity, were also recorded. Living as a couple was determined by the civil status reported by the individuals: married or living together. 

The control group included blood donors who fulfilled the national regulations: age ≥ 18 and ≤65 years, weight ≥50 kg, no tattooing or body piercing, no usage of illegal drugs, and no background of risky sexual practices or infectious diseases, inter alia [10]; and they were enrolled during the same period as patients with respiratory allergic diseases were. 

Loss of interest in sex

Loss of interest in sex was determined through item number 21 of the Beck Depression Inventory (BDI) II with the following options: 0) I have not noticed any current change in my interest in sex; 1) I am less interested in sex than I used to be 2) I am much less interested in sex and 3) I have completely lost my interest in sex. In the present study, the loss of interest in sex was assessed when the patient answered affirmatively to any of the options 1 to 3. The instrument was applied at the time of establishing the diagnosis of allergic disease.

Allergic asthma and allergic rhinitis

Asthma was defined as a personal history of wheezing, dyspnea, cough, and feeling of thoracic oppression, variable in time and intensity [11,12], together with forced spirometry, which demonstrated reversible obstruction of aerial flux after administration of a short-action bronchodilator; only those patients with symptoms compatible with asthma were susceptible to evaluation through respiratory function tests. Allergic rhinitis was defined as the presence of aqueous rhinorrhea, nasal obstruction, nasal itching, and sneezing induced by aeroallergen exposure [13]. Finally, allergic status was determined by at least one positive cutaneous test for any tested allergen.

Physical activity and body mass index

Conventionally, physical activity was assessed through an affirmative answer to the following question: “Do you practice physical activities such as running, swimming, ball games, or going to the gym for at least 30 minutes?” Body mass index (BMI) was obtained by dividing the body weight in kilograms by the square of the body height in meters. Excess weight was determined as the presence of overweight (BMI ≥25 and <30 kg/m^2^) or obesity (BMI ≥30 kg/m^2^).

Respiratory function tests

Forced spirometry was performed using Master Screen-Body PFT equipment (Jaeger®, Care Fusion, Baesweiler, Germany). All patients practiced the maneuvers in the mornings, with a maximum of eight attempts or until at least three curves were obtained according to acceptable and reproducible criteria. Thereafter, patients received 400 µg of salbutamol sulfate or 40 µg of ipratropium bromide through a spacer; after 15 to 20 minutes, forced spirometry maneuvers were repeated. A short-action bronchodilator test was considered positive when forced expiratory volume (FEV1) improved by more than 12% and more than 200 mL [14].

Skin testing technique

Aeroallergen sensitization was determined by the skin prick test; a total of 40 allergens (glycerinated and concentrated 1:20 weight/volume, not standardized) were tested. Several local pollen, fungal spores, and indoor allergens were included, and histamine and glycerin were used as positive and negative controls, respectively. Skin testing was performed according to international guidelines [15].

Statistical analysis

To obtain the prevalence, the number of subjects without interest in sex (presence of the event) was divided by the total number of subjects in each group. Continuous variables were compared with the Student-t or Mann-Whitney-Whitney U tests according to their distribution; categorical variables were compared through the chi-square test or Fisher’s exact test. Mean comparisons among asthma, allergic rhinitis, and control groups were assessed by ANOVA and Scheffé's post hoc test. To identify factors associated with the loss of interest in sex in patients with allergic asthma or allergic rhinitis, the control group was used as a reference. Initially, odds ratios and 95% confidence intervals (CIs) were calculated in 2 x 2 tables (univariate analysis). Later, in a stratified analysis, the odds ratio was calculated and adjusted by the Mantel-Haenszel method to search for an association between asthma or allergic rhinitis and loss of interest in sex, adjusted for possible confounding factors, viz., age, sex, living together, alcohol consumption, smoking, physical activity and overweight. Finally, multivariate models with binary logistic regression were performed, where the dependent variable was the loss of interest in sex and the independent variable was the presence of allergic asthma or rhinitis. The same variables used in the stratified analysis were introduced in multivariate analysis, and p ≤0.05 was considered statistically significant. An IBM® SPSS® Statistics 20 (IBM Corp., Armonk, NY, USA) program was used for data analysis.

## Results

Asthma subjects were older than the subjects in the control and allergic rhinitis groups (39.0 ± 14.0 vs 31.9 ± 10.6 or 32.4 ± 10.4, p <0.001, respectively), while no significant differences were found between the allergic rhinitis and control groups. The frequencies of living together (63.4%) and excess weight (72%) were higher in the asthma group than in the control group (45.9%). On the other hand, the control group revealed a higher frequency of smoking (15.6%), alcohol consumption (48.6%), and physical activity (72.5%).

If we considered the three groups as a combined one (n = 402), 153 patients (38.1%) denoted loss of interest in sex. The highest prevalence was observed in the asthma group (48.8%), followed by the allergic rhinitis group (39.5%) and y the control group (20.2%). The prevalence of the loss of interest in sex was 3.8 times higher in the asthma group (95% CI 2.2 to 6.6) and 2.6 times higher in the allergic rhinitis group (95% CI 1.4 to 4.6) than in the control group (p < 0.001 and 0.001, respectively).

Univariate analysis revealed that in the 402 subjects studied, being ≥ 40 years, being women, having a partner or spouse, a lack of physical activity, and being overweight were factors that increased the loss of interest in sex. On the other hand, tobacco or alcohol consumption decreased this probability (Table 1).

**Table 1:** Factors associated with the loss of interest in sex. OR: odds ratio of prevalence. The odds ratios for loss of interest in sex were calculated within the exposed and unexposed groups, respectively. Reference group: OR = 1. Comparison of proportions of two independent groups using the chi-square test.

	Loss of interest in sex (Dependent variable)			
Independent variable	Yes n = 153	No n = 249	OR (95% CI)	p
Age, n (%)				
≥ 40 years, n = 139	72 (51.8)	67 (48.2)	2.4 (1.6 - 3.7)	< 0.001
< 40 years, n = 263	81 (30.8)	182 (69.2)	1	
Sex, n (%)				
Female, n = 276	129 (46.7)	147 (53.3)	3.7 (2.3 - 6.2)	< 0.001
Male, n = 126	24 (19.0)	102 (81.0)	1	
Married or living together, n (%)				
Yes, n = 213	111 (52.1)	102 (47.9)	3.8 (2.5 - 5.9)	< 0.001
No, n = 189	42 (22.2)	147 (77.8)	1	
Current smoking, n (%)				
Yes, n = 39	8 (20.5)	31 (79.5)	0.4 (0.2 - 0.9)	0.02
No, n = 363	145 (39.9)	218 (60.1)	1	
Alcohol consumption, n (%)				
Yes, n = 119	31 (26.1)	88 (73.9)	0.5 (0.3 - 0.7)	0.001
No, n = 283	122 (43.1)	161 (56.9)	1	
Physical activity, n (%)				
No, n = 156	69 (44.2)	87 (55.8)	1.5 (1.01 - 2.3)	0.04
Yes, n = 246	84 (34.1)	162 (65.9)	1	
Excess weight, n (%)				
Yes, n = 261	110 (42.1)	151 (57.9)	1.7 (1.1 - 2.6)	0.02
No, n = 141	43 (30.5)	98 (69.5)	1	

Table 2 shows a stratified analysis where a significant association, in risk terms, was observed between asthma and loss of interest in sex, after adjusting for the following variables: age, sex, having a partner or spouse, alcohol consumption, smoking, physical activity, and overweight. Similar results were obtained related to the association of allergic rhinitis and the loss of interest in sex (Table 3), after adjusting for the same variables.

**Table 2:** Adjusted association by stratified analysis between asthma and loss of interest in sex. ORE: Odds ratio of prevalence calculated within each stratum, ORMH: Odds ratio obtained by the Mantel-Haenszel method. Proportions for two independent groups were compared by the chi-square test or Fisher's *exact test when the expected frequencies were < 5.

	Loss of interest in sex				
	Yes	No	OR_E_ (95% CI)	p	OR_MH_ (95% CI)
Age					
≥ 40 years					3.4 (1.9 - 6.0)
Asthma, n = 74	44 (59.5 %)	30 (40.5 %)	4.4 (1.7 - 11.6)	0.002	
Control, n = 28	7 (25.0 %)	21 (75.0 %)	1		
< 40 years					
Asthma, n = 90	36 (40.0 %)	54 (60.0 %)	2.9 (1.5 - 6.0)	0.002	
Control, n = 81	15 (18.5 %)	66 (81.5 5)	1		
Sex					2.8 (1.6 - 5.0)
Male					
Asthma, n = 33	11 (33.3 %)	22 (66.7 %)	4.4 (1.5 - 13.4)	0.006	
Control = 59	6 (10.2 %)	53 (89.8 %)	1		
Female					
Asthma, n = 131	69 (52.7 %)	62 (47.3 %)	2.4 (1.2 - 4.7)	0.01	
Control, n = 50	16 (32.0 %)	34 (68.0 %)	1		
Married or living together					
Yes					3.3 (1.9 - 5.9)
Asthma, n = 104	64 (61.5 %)	40 (38.5 %)	3.7 (1.8 - 7.7)	< 0.001	
Control, n = 50	15 (30.0 %)	35 (70.0 %)	1		
No					
Asthma, n = 60	16 (26.7 %)	44 (73.3 %)	2.7 (1.02 - 7.2)	0.04	
Control = 59	7 (11.9 %)	52 (88.1 %)	1		
Alcohol consumption					
Yes					3.1 (1.7 - 5.4)
Asthma, n = 33	14 (42.4 %)	19 (57.6 %)	7.1 (2.2 - 22.4)	< 0.001	
Control, n = 53	5 (9.4 %)	48 (90.6 %)	1		
No					
Asma, n = 131	66 (50.4 %)	65 (49.6 %)	2.3 (1.2 - 4.5)	0.01	
Control, n = 56	17 (30.4 %)	39 (69.6 %)	1		
Current smoking					
Yes					3.6 (2.1 - 6.3)
Asthma, n = 10	4 (40.0 %)	6 (60.0 %)	5.0 (0.7 - 34.9)	0.15*	
Control, n = 17	2 (11.8 %)	15 (88.2 %)	1		
No					
Asthma, n = 154	76 (49.4 %)	78 (50.6 %)	3.5 (2.0 - 6.3)	< 0.001	
Control, n = 92	20 (21.7 %)	72 (78.3 %)	1		
Physical activity					
Yes					3.5 (2.0 - 6.2)
Asthma, n = 90	42 (46.7 %)	48 (53.3 %)	4.4 (2.2 - 9.2)	< 0.001	
Control, n = 79	13 (16.5 %)	66 (83.5 %)	1		
No					
Asthma, n = 74	38 (51.4 %)	36 (48.6 %)	2.5 (1.0 - 6.1)	0.05	
Control, n = 30	9 (30.0 %)	21 (70.0 %)	1		
Excess weight					
Yes					3.8 (2.2 - 6.7)
Asthma, n = 118	64 (54.2 %)	54 (45.8 %)	4.2 (2.2 - 8.0)	< 0.001	
Control, n = 77	17 (22.1 %)	60 (77.9 %)	1		
No					
Asthma, n = 46 Control, n = 32	16 (34.8 %) 5 (15.6 %)	30 (65.2 %) 27 (84.4 %)	2.9 (0.9 - 8.9) 1	0.06	

**Table 3:** Adjusted association by stratified analysis between allergic rhinitis and loss of interest in sex. AR: Allergic rhinitis, ORE: Odds ratio of prevalence calculated within each stratum, ORMH: Odds ratio obtained by the Mantel-Haenszel method. Proportions for two independent groups were compared by the chi-square test or Fisher's *exact test when the expected frequencies were < 5.

	Loss of interest in sex				
	Yes	No	OR_E_ (95% CI)	p	OR_MH_ (95% CI)
Age					
≥ 40 years					2.6 (1.4 - 4.6)
AR, n = 37	21 (56.8 %)	16 (43.2 %)	3.9 (1.3 - 11.5)	0.01	
Control, n = 28	7 (25.0 %)	21 (75.0 %)	1		
< 40 years					
RA, n = 92	30 (32.6 %)	62 (67.4 %)	2.1 (1.05 - 4.4)	0.04	
Control, n = 81	15 (18.5 %)	66 (81.5 %)	1		
Sex					1.9 (1.05 - 3.6)
Male					
RA, n = 34	7 (20.6 %)	27 (79.4 %)	2.3 (0.7 - 7.5)	0.22*	
Control = 59	6 (10.2 %)	53 (89.8 %)	1		
Female					
RA, n = 95	44 (46.3 %)	51 (53.7 %)	1.8 (0.9 - 3.8)	0.10	
Control, n = 50	16 (32.0 %)	34 (68.0 %)	1		
Married or living together					2.8 (1.5 - 5.1)
Yes					
RA, n = 59	32 (54.2 %)	27 (45.8 %)	2.8 (1.3 - 6.4	0.01	
Control, n = 50	15 (30.0 %)	35 (70.0 %)	1		
No					
RA, n = 70	19 (27.1 %)	51 (72.9%)	2.8 (1.1 - 7.2)	0.03	
Control = 59	7 (11.9 %)	52 (88.1 %)	1		
Alcohol consumption					
Yes					2.2 (1.2 - 4.0)
RA, n = 33	12 (36.4 %)	21 (63.6 %)	5.5 (1.7 - 17.5)	0.002	
Control, n = 53	5 (9.4 %)	48 (90.6 %)	1		
No					
RA, n = 96	39 (40.6 %)	57 (59.4 %)	1.6 (0.8 - 3.2)	0.21	
Control, n = 56	17 (30.4 %)	39 (69.6 %)	1		
Current smoking					
Yes					2.5 (1.4 - 4.5)
RA, n = 12	2 (16.7 %)	10 (83.3 %)	1.5 (0.2 - 12.5)	0.99*	
Control, n = 17	2 (11.8 %)	15 (88.2 %)			
No			1		
RA, n = 117	49 (41.9 %)	68 (58.1 %)	2.6 (1.4 - 4.9)	0.002	
Control, n = 92	20 (21.7 %)	72 (78.3 %)	1		
Physical activity					
Yes					2.5 (1.4 - 4.4)
RA, n = 77	29 (37.7 %)	48 (62.3 %)	3.1 (1.4 - 6.5)	0.003	
Control, n = 79	13 (16.5 %)	66 (83.5 %)	1		
No					
RA, n = 52	22 (42.3 %)	30 (57.7 %)	1.7 (0.7 - 4.4)	0.27	
Control, n = 30	9 (30.0 %)	21 (70.0 %)	1		
Excess weight					
Yes					2.8 (1.5 - 5.1)
RA, n = 66	29 (43.9 %)	37 (56.1%)	2.8 (1.3 - 5.7)	0.005	
Control, n = 77	17 (22.1 %)	60 (77.9 %)	1		
No					
RA, n = 63	22 (34.9 %)	41 (65.1 %)	2.9 (1.0 - 8.6)	0.05	
Control, n = 32	5 (15.6 %)	27 (84.4 %)	1		

In multivariate analysis after statistical adjustment, having asthma (OR =2.4, 95% CI 1.3 to 4.5), being female (OR =3.4, 95% CI 1.7 to 6.6) and having a partner or spouse (OR =4.4, 95% CI 2.5 to 8.0) were risk factors for the loss of interest in sex (Model II, Table 4).

**Table 4:** Multivariate analysis of the association between asthma with loss of interest in sex. OR calculated by logistic regression. Reference category: OR = 1. Methods of entering variables into the models: Model I: Enter, Model II: Forward Conditional. ORs are not calculated for variables excluded from Model II.

	Model I			Model II		
	OR	95% CI	p	OR	95% CI	p
Group						
Asthma	2.0	1.1 - 3.9	0.03	2.4	1.3 - 4.5	0.005
Control	1			1		
Age						
≥ 40 years	1.6	0.9 - 2.9	0.12	---	----	0.08
< 40 years	1					
Sex						
Female	3.2	1.6 - 6.6	0.002	3.4	1.7 - 6.6	< 0.001
Male	1			1		
Married or living together						
Yes	3.8	2.1 - 7.0	< 0.001	4.4	2.5 - 8.0	< 0.001
No	1					
Current smoking						
Yes	0.6	0.2 - 1.6	0.31	---	----	0.35
No	1					
Alcohol consumption						
Yes	0.8	0.4 - 1.6	0.48	---	----	0.36
No	1					
Physical activity						
Yes	0.9	0.5 - 1.6	0.70	---	----	0.71
No	1					
Excess weight						
Yes	2.0	1.03 - 3.9	0.04	---	----	0.052
No	1					

Moreover, another multivariate analysis also identified the following variables as risk factors related to loss of interest in sex, when allergic rhinitis patients were compared to patients in the control group: having allergic rhinitis (OR = 2.1, 95% CI 1.1 to 3.9), being female (OR =3.7, 95% CI 1.8 to 7.6), and having a partner or spouse (OR =3.2, 95% CI 1.7 to 5.9) (Model II, Table 5).

**Table 5:** Multivariate analysis of the association between allergic rhinitis with loss of interest in sex. OR calculated by logistic regression. Reference category: OR = 1. Methods of entering variables into the models: Model I: Enter, Model II: Forward Conditional. ORs are not calculated for variables excluded from Model II.

	Model I			Model II		
	OR	95% CI	p	OR	95% CI	p
Grupo						
Allergic rhinitis	1.9	0.95 - 3.6	0.07	2.1	1.1 - 3.9	0.03
Control	1			1		
Age						
≥ 40 years	1.9	0.9 - 3.8	0.08	---	----	0.06
< 40 years	1					
Sex						
Female	3.7	1.7 - 7.9	0.001	3.7	1.8 - 7.6	< 0.001
Male	1			1		
Married or living together						
Yes	2.9	1.5 - 5.4	0.001	3.2	1.7 - 5.9	< 0.001
No	1			1		
Current smoking						
Yes	0.4	0.1 - 1.2	0.09	---	----	0.11
No	1					
Alcohol consumption						
Yes	0.99	0.5 - 2.1	0.99	-- -	----	0.49
No	1					
Physical activity						
Yes	0.7	0.4 - 1.4	0.30	---	----	0.31
No	1					
Excess weight						
Yes	1.1	0.6 - 2.2	0.72	---	----	0.52
No	1					

## Discussion

According to our results, the loss of interest in sex is highly prevalent in allergic asthma and rhinitis patients. Additionally, being female, having a partner, and having asthma or allergic rhinitis were factors associated with an increased probability of developing a loss of interest in sex. Almost half of the patients with allergic asthma and 40% of those with allergic rhinitis showed a loss of interest in sex, with a significantly higher prevalence than in the control group (20%); in general, this fact was notable in women (47%), compared to men (19%); similar findings have been reported in previous studies [7-9]. In contrast, it is important to consider that previous trials examined the loss of interest in sex for at least three months during the previous year, whereas, in our case, this variable was measured through one of the Beck Depression Inventory items, which inquired about depression symptoms two weeks before the test. Nonetheless, our study reveals two facts: one, patients with allergic respiratory diseases lose interest in sex with a higher frequency than non-allergic patients, and two, women are affected more by this problem than men.

A complex network of interactions among the central nervous, endocrine and immune systems seems to regulate the association between allergic inflammation and the loss of interest in sex. Experimental models in rats with allergic rhinitis have demonstrated an inflammatory response of the hippocampus, confirmed by the increased production of proinflammatory mediators after ovalbumin challenge models. Such changes could be expressed as abnormal behavior and negative emotions [16]. In particular, patients with symptoms of depression have shown high cytokine concentrations, including IL-6, TNF-α, IL-10, and IL-13, but decreased interferon-y levels, compared to subjects without symptoms of depression [17]. Notably, cytokine patterns seem to have different effects depending on sex; depressed women are likely to express higher quantities of IL-6 and leptin than men [18]. On the other hand, psychological stress can intensify allergic diseases by the stimulation of corticotropin-releasing hormone, neurotensin, and substance P. All these directly activate mast cells and ease proinflammatory cytokine production [19]. In our study, all patients with allergic asthma and allergic rhinitis shared allergic sensitization as a common feature. This event allowed us to suppose that typical cytokines in this group of patients (IL-4, IL-5, IL-13, inter alia) could be involved in the loss of interest in sex, but it might not be so. In any case, other kinds of auxiliary lymphocytes could be involved, such as Th-17 and its main metabolites, IL-17, or IL-6, when an increase in allergic diseases is reported [20,21]; thus, they might play a relevant role in the loss of interest in sex.

Apart from allergic asthma or allergic rhinitis as factors associated with the loss of interest in sex, two more elements were reported: living with a partner or being female. It has been demonstrated that married men and women have a lower risk of sexual problems than non-married, divorced, or separated men and women [22]. Another study confirmed that women had a higher loss of interest in sex than men; however, living without a partner acted as a protective factor for the loss of interest in sex [9]. The results of these studies differ completely from ours, although it is important to emphasize that we inquired about coexistence with a partner and not about the civil status of the subjects, as in previous reports. Several factors contribute to understanding the cause of the loss of interest in sex, including falling in love, lack of communication, years of living with a partner, rejection by a partner, and a power struggle [23].

The cross-sectional design of this study is one of its main limitations, wherein an interview detected the loss of interest in sex in the previous two weeks. On the other hand, even when allergic asthma or rhinitis diagnosis was obtained through clinical parameters, it should be underlined that both problems are chronic; consequently, it is very difficult to determine whether losing interest in sex was a recent event or appeared during the evolution of the allergic disease. Moreover, a detailed assessment of the patients related to the presence of organic diseases such as cardiac disease, renal disease, or hepatic insufficiency was not performed. Additionally, the quantification of serum levels of some male and female hormones was beyond the scope of the present study. From a psychosocial perspective, we did not inquire about any detailed problems regarding the relationship, partner communication, or their difficulties in being alone. Additionally, the role that allergic sensitization to seasonal and perennial allergens played in the loss of interest in sex was also not analyzed. Finally, sexual preferences or the frequency of sexual relations among the participants were not questioned.

## Conclusions

Allergic asthma and rhinitis are closely related to the loss of interest in sex, with a prevalence in almost half of the patients with the former and 40% of patients with the latter. Other associated factors identified independently were being female and living with a partner. It is advisable for the medical personnel treating patients with allergic asthma or rhinitis to intentionally identify the loss of interest in sex and other sexual problems and provide suggestions to improve them - in the best case, offer them help with specialized psychological care.
